# Improving the Health of Emerging Adult Gamers—A Scoping Review of Influences

**DOI:** 10.3390/nu14112226

**Published:** 2022-05-26

**Authors:** David Micallef, Lukas Parker, Linda Brennan, Bruno Schivinski, Michaela Jackson

**Affiliations:** School of Media and Communication, Royal Melbourne Institute of Technology, Melbourne 3000, Australia; lukas.parker@rmit.edu.au (L.P.); linda.brennan@rmit.edu.au (L.B.); bruno.schivinski@rmit.edu.au (B.S.); michaela.jackson@rmit.edu.au (M.J.)

**Keywords:** nutrition, diet, online games, gaming, eSports, video games, young adults, social marketing

## Abstract

Emerging adults (EAs), defined as adults aged 18 to 25, remain a difficult group to engage in healthy behaviours (including positive dieting and eating patterns). The environmental elements that influence the health behaviours of EAs have been studied. However, the literature is mixed on how online game environments, including eSports and game streaming, can be used to positively engage EAs. In this scoping review, we identified and analysed research on online games, EAs, and dietary patterns to create a behavioural ecological map of influences that intersect with EAs through online games. In total, 75 studies were found, identifying 23 influences that intersect with EAs through their online game use. ESports organisations, eSports athletes, and content creators may be areas of future research (and intervention) as these factors could positively influence the dietary behaviours of EAs (through online games).

## 1. Introduction

Emerging adulthood, defined as the life stage between 18 and 25 [1], is a critical life stage to address nutrition-related behaviours. This life stage is critical for the development of lifelong health habits that can prevent chronic diseases later in life [2,3]. It is also a stage when emerging adults (EAs) go through several life transitions, e.g., increased autonomy, changes in their physical environments, as well as changes to their influence networks, as they move out of their parental homes and enter new work and study environments [1,3,4]. During this period, EAs reduce their levels of physical activity in relation to their adolescence [4,5]; they are more likely to eat fewer fruits and vegetables and consume more energy-dense nutrient-poor (EDNP) foods [6]. EAs are also a lucrative target for companies looking to market EDNP foods, such as fast foods and sugar-sweetened beverages [3]. It is important to address the health behaviours of EAs to deal with long-term chronic health conditions related to poor dietary behaviours [7].

EAs are also the largest consumers of online games of any age group [8]. Online games have been linked with negative health impacts on nutritional outcomes as a result of increased consumption of savoury snacks and sugar-sweetened beverages during gameplay or an increased likelihood of skipping meals [9]. An association between video games and an increased risk of a higher BMI [9], lower general health status, and higher levels of sedentary behaviour were also found [10]. Stress markers during video gameplay have been shown to increase the number of calories consumed, with a preference for fatty and sweet foods [11]. Whilst research currently shows a link between video gameplay and negative health outcomes, the use of online games as a potential mechanism for engaging EAs in healthy behaviours is an area that is currently under-researched [12].

The purpose of this scoping review was to gather, review, and summarize the academic literature regarding the engagement of EAs in online games and identify factors that may support positive engagement in diet interventions.

### 1.1. Understanding the Behavioural Ecology of EAs within Online Games

EAs’ lived experiences influence their health-related behaviours. Understanding the context in which behaviours occur is important for developing interventions that will improve health outcomes for EAs [13]. Recently, researchers introduced behavioural ecology concepts to the nutrition and public health fields [14]. Relatively recently, social marketers have begun to use behavioural ecological models (BEMs) to understand what influences the behaviours of respective target audiences [15,16], as well as the behavioural ecologies that their audiences exist within [14,17]. BEMs provide a framework for understanding these influences as they seek to map the multiple actors, interactants, and actions within an individual’s behavioural ecology [18]. There are many extant models used to understand BEMs, which include multiple variables and influences, including the sociocultural ecological systems model [19,20] and the determinants of health model [19].

A four-level BEM [21] was previously used to understand the influences on the physical activities and dietary behaviours of EAs [12]. This included (1) sociocultural influences, such as culture, social norms, and gender; (2) community influences, such as media, social media, and micro-celebrities; (3) local influences, e.g., schools, workplace, and physical environments; and (4) individual influences, such as peers, intimate partners, home environments, and parents. Recently Micallef, Brennan, Parker, Shivinski and Jackson’s [12] research identified systemic impacts on the physical activities and dietary behaviours of EAs, such as the impacts of peer influence and social media. However, they found that there was little research into where online games fit into the health behaviour ecologies of EAs, despite games being such significant components of EAs’ lives. The current study addresses the call for research to understand influences within an online game’s behavioural ecology and the impact that these influences may have on the physical activities and dietary behaviours of EAs [13]. Understanding the relationship between physical activity and diet behaviour is paramount to informing clinical practices and health campaigns and, therefore, helps EAs to better understand the implications of diet on their overall health [13].

### 1.2. EAs Enter a Global Behavioural Ecology When Playing Online Games

Playing online games has become a popular pastime for EAs [9,10,22]. For example, in Australia, EAs are some of the largest consumers of online games, with 82% of 15–24 year-olds playing video games, with an average play time of 89 min per day [8]. The COVID-19 pandemic has also seen an increase in the use of online games [23], with EAs turning to online games as a way to maintain relationships with peers and create new connections [22]. Advances in internet technologies have seen video games move from localised activities with friends and family to global activities, where EAs engage in gameplay daily with players from within their regions or from across the world [24,25]. Online games range from strategy games, first-person shooters, and sports games; a player may team up to play with a small group of players or play in a large-scale multiplayer online battle arena (MOBA). There are also massive multiplayer online role-playing games (MMORPG) where players enter a virtual world with thousands of players playing simultaneously. From a BEM perspective, EAs interact with a range of potential influences by playing online games (see Micallef et al., 2021 for an overview). Whilst a link was shown in previous research between playing online games and negative nutritional outcomes, to the best of our knowledge, there is no research to show what role these interactions may or may not have on the health behaviours of EAs.

The behavioural ecology of the online game world extends beyond the other players who users might interact with during the game. Online game streaming services, such as Twitch, have become media platforms where content creators stream hours of content each day to thousands of people. Viewers seek game-playing tips, share strategies, and engage with online peers. A recent study by de Wit, et al. [26] found that 61% of EAs viewed Twitch for more than 7 h a day, with 48% of participants viewing streams 6–7 days per week. Electronic sports (eSports) have created competitive and professional elements to online gameplay that provide engagement for EAs outside of direct play [27]. The professionalisation of eSports includes teams, leagues, and tournaments being set up globally and being recognised as an official sport in many countries; it is predicted that eSports will become an Olympic sport [28]. For EAs, online games are activities that are a part of day-to-day life just as much as social media. This extension of online games in adjoining activities, such as streaming and eSports, further highlights the extensive behavioural ecology that EAs participate in, underscoring the importance of understanding whether this behavioural ecological system can be used to impact health behaviours of EAs.

### 1.3. The Current Study

This study sought to extend previous work [12] in order to more deeply examine the online game behavioural ecologies in which EAs engage. We conducted a scoping review to understand the behavioural ecological influences that EAs engage in through online games. A scoping review is an appropriate methodology to use when mapping existing research within a field, as it allows for a descriptive overview of the research [29]. The scarcity of research into the health behaviour ecologies of EAs in online games limits the ability to synthesize the available research into online game influences. Although previous reviews were conducted [10,11,13], to the best of the authors’ knowledge this is the first review that aims to create an ecological map of influences on the health behaviours of EAs through online games. The following research objective guided our scoping review:

- Research objective (RO): to identify (in the literature) the potential behavioural influences of EAs through online games and in related activities, such as eSports and streaming.

Identifying potential influences according to the research objective and the development of a behavioural ecological ‘map’ of these influences (according to the BEM) is a useful artefact to guide future research in this field and to support the development/testing of interventions that seek to impact the healthful behaviours of EAs.

## 2. Materials and Methods

The Arksey and O’Malley [29] five-step procedure was used for this scoping review: (i) identify the research question; (ii) identify relevant studies; (iii) select studies using the PRISMA protocol; (iv) chart the data; and (v) collate, summarize, and report the results.

In Step 1, the research objective was developed to guide the research process and a search strategy was developed to guide the literature search. In Step 2, two search strings were used to identify literature related to online games and emerging adults, as well as online games and physical activity or nutrition. A prior scoping review conducted by Micallef, Brennan, Parker, Schivinski and Jackson [12] combined both search strings and identified a lack of studies focusing on nutrition and online games. Based on this previous study, a decision was made to include physical activity in this search. A full list of search terms is provided in Appendix A. The following six databases were searched identifying a total of 6139 articles: EBSCO (e.g., AMED, Business Source Complete, Academic Source Complete, and SPORTDiscus), CINAHL, OVID, Science Direct, Web of Science, and IEEE Xplore. The databases were chosen because they have cross-sections of social sciences and technological literature. The search was limited to the title, abstract, and keywords. As online games and eSports have grown in recent years, a decision was made to restrict the search from 2010 to 2022 to identify the most recent literature on the topic. Only articles available in English and the full text were searched.

In Step 3, duplicates were removed, and the title and abstract were reviewed to identify the literature relevant to the study question. Articles that were not in English, but which still appeared in search results (*n* = 21) and articles that did not meet the study criteria (*n* = 5991) were removed. For example, articles related to the use of games in the treatment of disease and articles that concentrated on children (or were not relevant to EAs) were excluded during this step. Studies were included if they were relevant to EAs or potentially identified influences in the online game behavioural ecology. A total of 107 articles were identified for the full-text review.

**Figure 1:**
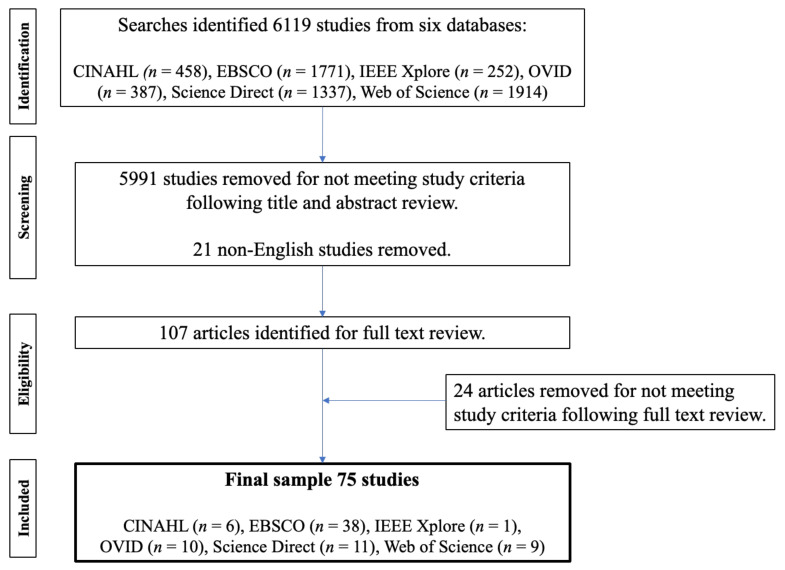
PRISMA flowchart for this study.

A full-text review of the 107 articles identified studies that were not relevant to the research objective. For example, Boulos and Yang [30], De Grove [31], Crowe [32], and Foley et al. [33] were removed as the focus was on children or adolescents. A final total of 75 studies were identified during this step. Figure 1 is the PRISMA flowchart for the selection of studies.

A quality assessment was undertaken of the articles using the Evans [34] hierarchy of evidence framework, as well as Brennan, Binney, Parker, Aleti and Nguyen [19]’s levels of evidence required for decision-making. Quality assessment was necessary due to studies being broad in terms of their study populations. The use of both studies allowed for an assessment based on the methodology using a known framework, as well as an assessment of the relevance of the study to EAs. The quality assessment rating was used when considering whether findings were relevant to EAs. No studies were excluded during the quality assessment process.

The full text of the identified studies was reviewed and the data were charted in Microsoft Excel (Step 4). The data extracted included the publication details (authors, journal, year of publication, keywords), abstract, study aims, methodology, country of study, population count, and demographics (age and gender or sex, where specified). The main findings of the study were extracted; the full text was reviewed to identify influences that may have had a role in influencing EAs through online games and related activities. Influences identified in any part of the paper were included.

In Step 5, a coding frame was developed based on the influences identified in the charting of data and grouped by the levels of the BEM. Sociocultural influences of the BEMs were not included in the coding frame as the study aimed to focus on identifying influences in online games that could be impacted directly by health and social marketing practitioners. Studies were analysed based on the identification of influences as well as the textual analyses of the results. Further analysis of the studies was conducted on the participant size, country of origin, age, and gender. Two investigators independently screened a selection (*n* = 10) of the included studies to confirm the analyses of the studies. All conflicts were discussed until a joint consensus was reached on the studies.

## 3. Results

**Figure 2:**
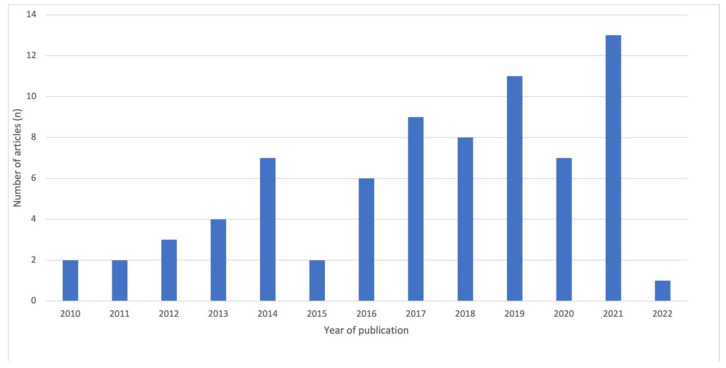
Year of publication analysis.

The scoping review identified a total of *n* = 75 studies that identified potential influences on the diet behaviours of EAs in the online game behavioural ecology. Most studies (*n* = 49; 65.3%) were published between 2017 and 2022, with the largest number of studies published in 2021 (*n* = 13; 17.3%). Figure 2 charts the number of studies by year and shows an increase in research on online games in the last five years.

Studies included a range of qualitative and quantitative methodologies including observations, interviews, surveys, and experiments. Literature reviews were also included as well as some perspective articles (*n* = 5; 6.6%) [27,35,36,37,38] due to their relevance to the research objective. For empirical studies (*n* = 61; 81.3%), participants were mostly recruited from the United States (*n* = 20; 32.7%), followed by the United Kingdom (*n* = 4; 6.6%), Spain (*n* = 3; 4.9%), Australia (*n* = 3; 4.9%), and Taiwan (*n* = 3; 4.9%). Seven studies (11.4%) included participants from multiple countries; the remainder (*n* = 21; 34.4%) were from individual countries.

Study populations ranged from *n* = 2 [39] to *n* = 11,018 [28] and included male, female, and non-binary genders. Participant ages ranged from 12 to 60+ for all, except for one empirical study including EAs. Kuukka, et al.’s [40] case study exploration of an online console game club (*n* = 8 participants, aged 26–28) was included as the participants reflected on their participation within a game club that they joined as EAs. The studies included in the scoping review and their key characteristics are provided in Table 1. Two empirical studies [11,41] did not list their population age ranges and are listed as “not stated” (N/S) in the table. Gan, Servio, Fewtrell and Wells [11] focused their study on “young men”; Evans, Evans, Shank and Fallon [41] conducted a virtual survey of Pokémon Go users, which other studies (e.g., [42,43]) showed EAs. Non-empirical studies (*n* = 14; 18.7%) were designated as “not applicable” (N/A) for participant ages but were relevant to emerging adulthood. For example, Bragg, et al. [44] was a perspective article that discussed the health implications of food and beverage marketing to youth through sports, including sports video games. Adachi and Willoughby [45] conducted a literature review on the link between playing video games and positive youth outcomes.

### 3.1. Mapping the Potential Influences in Online Games

**Figure 3:**
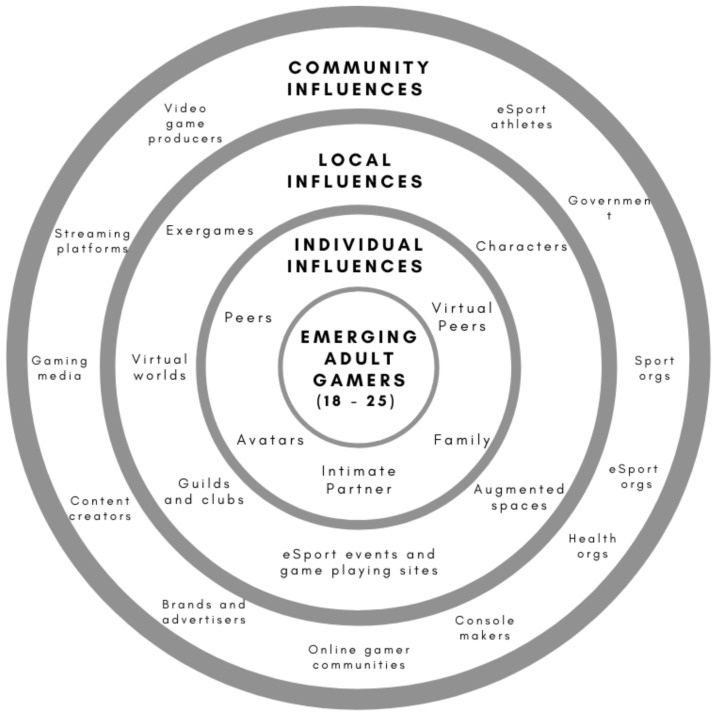
Behavioural ecological map of influences that intersect with EAs in online games.

To answer the research objective, the identified influences within the literature were mapped according to the BEM. A total of *n* = 23 influences were identified in the research. These influences intersect with EAs when they play online games or participate in eSports or online game streaming. Based on the BEM, influences included *n* = 5 (21.7%) influences at the individual level of the BEM, *n* = 6 (26.1%) influences at the local level, and *n* = 12 (52.2%) influences at the community level. Figure 3 charts the behavioural ecological map of influences of EAs based on the influences identified in the scoping review.

The studies identified in the scoping review were more likely to identify influences at the local level of the BEM than any other level. Out of the 75 studies in the scoping review, local influences were identified in *n* = 46 studies. Influences at this level included an EA gamer’s interaction with his/her virtual environment, characters within this world, or the cross-over of real and virtual worlds through augmented games, exergames, or events and clubs focused on gamers. Community influences were identified in *n* = 26 studies and included the owners of virtual worlds and game platforms, eSports organisations, content creators and professional players, as well as governments, sports, and commercial organisations who have an interest in the online game environment. Individual influences were identified in *n* = 42 and included peers, family, and interactions with in-game avatars. This suggests that current research focuses more heavily on gamers’ interactions with their virtual environments than on any other influence level. Table 2 provides definitions for influences at each BEM level and the relevant references for the definitions, where defined in the research.

### 3.2. Game Ecology Influences on EA Health

An analysis of the studies identified in the scoping review provided some indicators as to the intersections of these influences regarding the health of EAs based on the three levels of the BEM explored in the review: community; local; and individual.

At the community level of the BEM, video game producers and console makers were identified as influences—as the ultimate owners, makers, and marketers of online game worlds. Video game producers have a role in influencing the health of players passively [10,11,58], through gameplay mechanics [99] and the development and release of exergames and serious games [39,53,68,75]. Due to the advancements in the internet and streaming technologies, there has been a boom in content creators who develop influencer communities around gameplay [98]. Regarding technological advancements, there have been increases in the development and visibility of eSports organisations, eSports athletes [29,57,90], and large communities of gamers and viewers around their sports. Moreover, eSports athletes, many of whom are EAs themselves, have been the subjects of research linking their health to game performance [71,89]. This growth involves online games becoming a part of marketing strategies for organisations targeting EAs [56]. Branding inside video games has been shown to increase affinity to a brand [59] and products that are detrimental to health have become commonplace in online games including tobacco [48,54], energy-dense nutrient-poor foods, and sugar-sweetened beverages [44]. For health and nutrition intervention, this suggests a potential opportunity through a range of media channels and influences at this level that are already being utilised by other organisations to engage EAs.

At the local level, the online and virtual worlds that EAs enter through online games can influence their real-world actions. Engaging in virtual worlds and characters can make gamers consider societal problems or help them problem-solve real-world issues [47,104]. Gamers who play virtual sports games are also more likely to engage in the sport in real life [45,97]. Augmented games, which embed virtual elements into the real environment, have been shown to increase physical activity [77,88,102], whilst eSports, which involve players and teams competing in virtual games, attract audiences in both virtual and physical spaces [89]. Online game clubs and guilds, which create hubs for players with shared game interests, foster the development of peer connections and allow gamers to expand their personal networks [40,45]. This shows that there is a vast local environment that EAs engage in through online games—both virtual and physical—that potentially impacts the health behaviours of EAs or can be used through health interventions to positively impact their behaviours. Recent research has identified the beneficial role that online third places have in engaging consumers [105], suggesting that there is a potential to explore the use of these spaces to engage EAs in healthy behaviours.

At the individual levels of BEMs, studies debunk the myth of the lonely gamer and find that gamers play with a range of real-life and virtual connections including peers, family, and intimate partners [25]. Whist online gameplay can be a form of escapism [52,58], gamers have a similar number of social connections as compared to non-game players [65]. For gamers, social elements are an essential part of gameplay [96,99] as they form mechanisms to find peers with similar interests [47,52]. The COVID-19 pandemic led to increasing blurred lines between real life and virtual networks; EAs used online games to maintain relationships with their current friendship networks and to create new relationships and networks online [22]. Previous research has already shown the importance of peer networks in influencing the diet behaviours of EAs [12], but there is little research to understand how game-related peer networks impact this behaviour.

New peer networks are not the only influences at the individual levels of BEMs. Avatars, the virtual representations of players inside game spaces, can impact levels of exercise in exergames [64,78]. Identification with a gamer’s avatar can also increase the risk of an internet gaming disorder [104]. Liew, Stavropoulos, Adams, Burleigh and Griffiths’ [81] study found a correlation between identification with a user’s avatar and the amount of time spent in a game to the detriment of real-life activities, such as physical activity. While links have been explored between avatars and physical activity levels, there is currently a gap in understanding whether avatars may play influential roles in other areas of health [104], such as nutrition.

## 4. Discussion, Limitations, and Implications for Future Practices

This scoping review indicates that EAs who engage in online games participate in substantial ecological influences at the three levels of the BEM explored. These influences were shown through various studies to impact the behaviours of EAs, including positive and negative health behaviours. Whilst EAs engage in behavioural ecologies differently from other life stages (due to a range of instabilities in their lives), consistent with the findings of Arnett [1], the behavioural ecology within online games may provide a stable avenue in which to engage EAs, to encourage positive health behaviours. For example, a national campaign targeting fruit and vegetable consumption in EAs may potentially engage with a national eSports tournament through sponsorship and advertising (community level), conduct activities at physical tournaments and specific gamer clubs (local level), and encourage team and peer competitions in the outcomes of the campaign (individual level).

### Limitations and Implications for Future Research and Practices

This is the first scoping review to map the influences of EAs in the online game behavioural ecology, guiding the further development of research and interventions to impact the health of EAs through this popular medium. Whilst the review identified *n* = 23 potential influences on the health behaviours of EAs at the community, local, and individual levels of BEMs, the studies identified in this scoping review did not directly assess the potential impact of these influences on the diet behaviours of EAs, providing a large scope for future research and the testing of dietary interventions through the online game behavioural ecology. For example, there is already a range of marketing avenues available through eSports, such as tournament and league sponsorships, physical and digital marketing, and product placements [27]. These avenues are being used by a range of organisations seeking to engage EAs [56], but have yet to be explored in the context of diet interventions for EAs. Further research could seek to identify whether food- and diet-related messaging through these channels impact EA behaviours and whether sponsorships and advertising through eSports may be viable channels to engage EAs on this topic.

Similarly, as eSports are becoming more professionalised, there is an increased focus on the link between physical health and in-game performances for eSports athletes. Improving health regimes, such as physical activity training and the use of nutritionists to plan meals, have become part of the training for eSports athletes due to the benefits of improving in-game focus [90]. Whilst there are aspirational elements for gamers in watching professional players play games and compete [24], the scoping review did not identify any research exploring whether the link between good health and in-game performance could be used to influence EA gamers. Just as health promotion has used traditional sports players as influencers in social marketing campaigns, there is a potential to explore whether the link between health and performance in eSports could be used as a motivator to engage casual EA gamers in positive health behaviours.

Content creators, or ‘streamers’, are the microcelebrities of the online game world and create communities where they engage with EAs but also allow EAs to create new peer networks. These content creators are integral parts of a media ecosystem that already succeeds in engaging hard-to-reach audiences, such as EAs, with entertaining content [38]. Research shows that streamers could positively impact the mental health of their viewers through their own mental health disclosures [106]; these channels are also being used for the promotion of products. However, there is little research to understand their potential impacts on influencing other health behaviours. Future research could explore whether streamers can influence positive dietary behaviours in their audiences, just as micro-celebrities in other target audience groups (e.g., the fashion and beauty industries) influence their respective communities [107].

## 5. Conclusions

To the authors’ knowledge, this is the first study to create a behavioural ecological map of influences that EAs intersect with when engaging in online games. The high levels of online game usage by EAs and the increase in viewership through eSports and streaming platforms make this behavioural ecology an important platform to engage and influence EAs at important life junctures for long-term health behaviours. The results of this scoping review provide an opportunity for researchers, health promotion agencies, and health practitioners to combat the worsening diet outcomes of EAs by delivering interventions through a behavioural ecology that EAs are already highly engaged in. The current use of this behavioural ecology for the promotion of products that worsen diet outcomes, such as the promotion of EDNP foods and sugar-sweetened beverages, further highlights the importance of exploring this ecology to improve health.

## Figures and Tables

**Table 1:** Characteristics of scoping review studies from the two independent literature searches.

Author	Year	Search Theme	Participants	Location	Age	Gender	Influences Identified
Padilla-Walker, et al. [46]	2010	Emerging adults	813	USA	18–26	M; F	Peers; family; intimate partners
Skoric and Kwan [47]	2011	Emerging Adults	385	Singapore	18–29	M; F	Peers; virtual peers; virtual worlds
Perez, et al. [48]	2012	Emerging Adults	1000	Australia	12–24	M; F	Brands and advertisers
Kontour [35]	2012	Emerging adults	N/A	N/A	N/A	M	Online gamer communities; content creators; video game publishers; avatars
Coyne, et al. [49]	2013	Emerging adults	N/A	N/A	N/A	N/A	Peers; Family
Schiano, Nardi, Debeauvais, Ducheneaut and Yee [25]	2014	Emerging Adults	2865	Worldwide	18–40	M; F	Peers; family; intimate partners; virtual peers
Bourgonjon [36]	2014	Emerging adults	N/A	N/A	N/A	N/A	Video game producers; government and policymakers;
Bean, et al. [50]	2016	Emerging adults	465	Worldwide	18–25	M; F	Peers
Millington [37]	2016	Emerging adults	N/A	N/A	N/A	N/A	Console makers; video game producers; family; government and policymakers; health organisations
Poppelaars, et al. [51]	2018	Emerging Adults	146	USA	20.21 (mean)	M; F	Health organisations; video game producers;
Peeples, et al. [52]	2018	Emerging adults	N/A	N/A	N/A	N/A	Virtual worlds; virtual peers; characters; avatars
Bragg, Roberto, Harris, Brownell and Elbel [44]	2018	Emerging adults	N/A	N/A	N/A	N/A	Brands and advertisers; video game producers; government; and policymakers
Nordby, et al. [53]	2019	Emerging adults	393	Norway	18–60	M; F	Video game producers
Kuukka, Uusiautti and Maatta [40]	2019	Emerging adults	8	Finland	26–28	M; Non-Binary	Peers; guilds and clubs
McDaniel and Forsyth [54]	2019	Emerging adults	N/A	N/A	N/A	N/A	Brands and advertisers; video game-playing sites
Chung, et al. [55]	2019	Emerging adults	N/A	N/A	N/A	N/A	Online gamer communities; government and policymakers; video game producers; streaming platforms; eSports organisations; eSports athletes
García and Murillo [28]	2020	Emerging adults	11,018	Spain	15+	M; F	Online gamer communities; streaming platforms; eSports organisations; sports organisations
Elasri-Ejjaberi, et al. [56]	2020	Emerging adults	1619	Spain	8–14 and 15–25	M; F	Online gamer communities; content creators; Streaming platforms; eSports organisations; Sports organisations; brands and advertisers
Kelly, et al. [57]	2021	Emerging adults	905	Australia	12–24 + parents (age NS)	M; F	Peers; Virtual peers; health organisations; video game producers
Bengtsson, Blackman, King, Østergaard, Bengtsson, Bom and Fynbo [22]	2021	Emerging adults	35	Denmark	16–19	F; M	Peers; virtual peers; parents
Hussain, et al. [58]	2021	Emerging adults	9	Pakistan	18–29	F	Avatars; Virtual peers; Virtual worlds
van Berlo, et al. [59]	2021	Emerging adults	81	Netherlands	18–30	M; F	Virtual worlds; brands and advertisers
Chan, Huo, Kelly, Leung, Tisdale and Gullo [9]	2022	Emerging adults	N/A	N/A	N/A	N/A	eSports organisations
Jin [60]	2010	PA and nutrition	75	USA	College students	M; F	Avatars; augmented spaces
Song, et al. [61]	2011	PA and nutrition	85	USA	College students	M; F	Avatar; Augmented spaces
Garn, et al. [62]	2012	PA and nutrition	30	USA	20.5 (mean)	M; F	Exergames; peers
Peng and Crouse [63]	2013	PA and nutrition	162	USA	18–23	M;F	Virtual peers; augmented spaces
Shaw [39]	2013	PA and nutrition	2	USA	23–34	F	Virtual worlds; characters; avatars; online gamer communities; video game media
Kastenmuller, et al. [64]	2013	PA and nutrition	147	UK	20–22 (mean across 3 studies)	M; F	Avatars
Kowert, et al. [65]	2014	PA and nutrition	2551	Germany	14+	M;F	Peers;
Vernadakis, et al. [66]	2014	PA and nutrition	232	Greece	18–20	M; F	Augmented spaces
Lyons, et al. [67]	2014	PA and nutrition	97	USA	18–35	M; F	Video game producers; augmented spaces
Kim, et al. [68]	2014	PA and nutrition	119	USA	18–42	M; F	Augmented spaces; exergames; avatars
Gan, Servio, Fewtrell and Wells [11]	2014	PA and nutrition	72	UK	NS	M	Virtual worlds
Peng, et al. [69]	2015	PA and nutrition	127	USA	18–25	M; F	Augmented spaces
Kakinami, et al. [70]	2015	PA and nutrition	829	Canada	24 (mean)	M; F	Exergames
Kari and Karhulahti [71]	2016	PA and nutrition	115	Worldwide	16–30+	M; F	eSports athletes
Henchoz, et al. [72]	2016	PA and nutrition	4933	Switzerland	18–25 (mean 19.95 at baseline)	M	Peers;
Nguyen, et al. [73]	2016	PA and nutrition	117	Taiwan	21–31	NS	Augmented spaces; exergames
Kim and Timmerman [74]	2016	PA and nutrition	47	USA	21.75 (mean)	M; F;	Avatars; video game producers;
Kaczmarek, Misiak, Behnke, Dziekan and Guzik [42]	2017	PA and nutrition	444	Poland	12–50	M; F	Augmented spaces; exergames
Said Vojciechowski, et al. [75]	2017	PA and nutrition	40	Brazil	18–30	M; F	Augmented spaces
Yang and Liu [43]	2017	PA and nutrition	262	USA	18–58	M; F	Peers; Virtual peers; augmented spaces;
Wong [76]	2017	PA and nutrition	644	Hong Kong	18–60 (majority 18–25)	M; F	Augmented spaces
Huang, et al. [77]	2017	PA and nutrition	113	Taiwan	20–24	M; F	Exergames;
Joo and Kim [78]	2017	PA and nutrition	124	South Korea	20–29	M; F	Avatars
Nigg, et al. [79]	2017	PA and nutrition	486	USA	28.6 (mean)	M; F	Augmented spaces;
Krittanawong, et al. [80]	2017	PA and nutrition	N/A	Worldwide	N/A	N/A	Augmented spaces; exergames
Adachi and Willoughby [45]	2017	PA and nutrition	N/A	N/A	N/A	N/A	Virtual peers; guilds and clubs; avatars; augmented spaces
Liew, et al. [81]	2018	PA and nutrition	121	Australia	18–29	M; F	Peers; avatars; virtual worlds;
Gabbiadini, et al. [82]	2018	PA and nutrition	981	USA	18+ (mean 32.55)	M; F	Augmented spaces; peers
Wattanapisit, et al. [83]	2018	PA and nutrition	26	Thailand	20–24	M; F	Augmented spaces
Marquet, et al. [84]	2018	PA and nutrition	74	USA	College students	M; F	Augmented spaces
Hallmann and Giel [27]	2018	PA and nutrition	N/A	N/A	N/A	N/A	eSports athletes; eSports organisations; eSports events and game-playing sites; sports organisations; government and policymakers
Williams and Slak-Valek [85]	2019	PA and nutrition	438	Worldwide	18+	M; F	Augmented spaces;
Huang, et al. [86]	2019	PA and nutrition	337	Taiwan	18+	M; F	Exergames;
Bock, et al. [87]	2019	PA and nutrition	189	USA	20–79	M; F	Virtual worlds; exergames;
Ni, et al. [88]	2019	PA and nutrition	65	Hong Kong	20.7 (mean)	M; F	Augmented spaces
Ekdahl and Ravn [89]	2019	PA and nutrition	N/A	Denmark	N/A	N/A	eSports athletes; eSports organisations; eSports events; virtual worlds
Pargman and Svensson [90]	2019	PA and nutrition	N/A	Sweden	N/A	N/A	eSports organisations; sports organisations; video game producers; virtual worlds
Faric, et al. [91]	2019	PA and nutrition	N/A	UK	N/A	N/A	Virtual worlds; exergames; peers
Frolich, et al. [92]	2020	PA and nutrition	230	USA	18–54	M; F; NB	Characters; augmented spaces
Soltani, et al. [93]	2020	PA and nutrition	76	France	19–30	M;F	Augmented spaces
Marello, et al. [94]	2020	PA and nutrition	130	USA	19–76	M; F	Augmented spaces; peers; family
Yan, et al. [95]	2020	PA and nutrition	288	USA	19.03 (mean)	M; F	Augmented spaces; exergames
Pelletier, Lessard, Piche, Tetreau and Descarreaux [10]	2020	PA and nutrition	N/A	N/A	N/A	N/A	eSports organisations; sports organisations; health organisations; government and policymakers
Laato, et al. [96]	2021	PA and nutrition	515	Finland	18+ (48% 18–25)	M;F	Peers; virtual peers; augmented spaces
Pereira, et al. [97]	2021	PA and nutrition	433	Portugal	18+ (median 22)	M; F	Virtual worlds
Yoganathan, et al. [98]	2021	PA and nutrition	15 (focus groups)	UK	19–30	M;F	Content creator; online gamer communities; virtual peers
Kim [99]	2021	PA and nutrition	258	USA	19+	M;F	Video game producers; virtual peers
Esteves, et al. [100]	2021	PA and nutrition	212	Spain	23–30	M; F	Peers; virtual peers
Wang and Skjervold [101]	2021	PA and nutrition	2191	Worldwide	5–67 (mean 24)	M; F	Augmented spaces; peers; virtual peers
Lee, et al. [102]	2021	PA and nutrition	N/A	N/A	N/A	N/A	Augmented spaces; virtual peers
Ketelhut, Martin-Niedecken, Zimmermann and Nigg [38]	2021	PA and nutrition	N/A	N/A	N/A	N/A	Augmented spaces; exergames; streaming; eSports organisations; content creator; eSports athletes
Evans, Evans, Shank and Fallon [41]	2021	PA and nutrition	N/S	Worldwide	N/S	N/S	Augmented spaces; peers; virtual peers

Abbreviations: N/A: element does not apply to the study; not stated: element not indicated in the study; M = male; F = female.

**Table 2:** Definitions of influences identified in the scoping review.

Influence	Definition	Reference
Micro-level (individual)		
Peers	A person in the same social group and/or demographic with which the person regularly interacts in real life as well as virtually.	[22]
Avatars	The virtual representation of a player in an online game space and/or community. This can either be pre-determined by the game or virtual space or highly customisable by the user.	[58]
Virtual peers	Peers that an individual only knows through a virtual environment, such as an online game or an online channel.	[22]
Family	A person or people with whom the individual is directly related to.	[12]
Intimate partner	A person the individual is romantically or sexually involved with.	[12]
Local influences		
Virtual worlds	The virtual game environment in which a user interacts. This can range from contained arenas, such as those in first-person shooter games, to extensive virtual worlds, such as those in strategy and role-player games.	[52]
Characters	The virtual characters in a game space that allow for interaction and progress the virtual story.	[52]
Augmented spaces	Physical spaces that are augmented through online games using a smartphone’s GPS and camera functions.	[42]
Exergames	Video games that require the player to conduct physical movements to participate in the game.	[93]
Guilds and clubs	Clubs for online gamers. These can be clubs centred around specific games for individual gamers to play together.	[40]
eSports events and video game-playing sites	Public, event-based game activity, including arena-style eSports events and smaller video game arcades and bars.	[90]
Community influences		
eSports athletes	Professional players who derive an income from playing online games competitively.	[90]
Video game producers	The publishers of online game content.	
Brands and advertisers	Organisations who target gamers through branding and advertising in online games and related channels.	[56]
eSports organisations	Organisations in eSports, including eSports teams, eSports leagues, and governing associations.	[90]
Console makers	Creators of video game consoles, such as Sony (PlayStation), Microsoft (Xbox), and Nintendo (Wii).	
Health organisations	Organisations whose primary missions are to improve the health of their communities.	
Streaming platforms	A platform that allows the live-sharing of gameplay and other media content, such as Twitch or YouTube. The platforms also allow for live interactions with viewers of the stream.	[24]
Content creators	They are also referred to as streamers; content creators create game-related media content through streaming platforms such as Twitch for their viewers.	[98]
Sports organisations	Traditional sports clubs and governing bodies.	[103]
Online gamer communities	General clubs and online communities where participants come together to discuss online games and potentially find virtual peers to play specific games or form guilds/clubs (See guilds and clubs).	[40]
Government and policymakers	Governing bodies and those involved in the development of government policies.	
Video game media	Traditional and online media focused on video games.	

## Appendix A. Search Terms

Search One (Games and Emerging Adults)

- Gaming OR “video game*” OR “computer game*” OR exergame OR “serious game*” OR gamification OR esport; AND
- “emerging adults” OR “young people” OR youth

Search two (games and health):

- Gaming OR “video game*” OR “computer game*” OR exergame OR “serious game*” OR gamification OR esport; AND
- “physical activity” OR exercise OR diet OR nutrition
